# Directional Modulation-Enhanced Multiple Antenna Arrays for Secure and Precise Wireless Transmission

**DOI:** 10.3390/s19224833

**Published:** 2019-11-06

**Authors:** Wei Zhang, Mingnan Le, Bin Li, Jun Wang, Jinye Peng

**Affiliations:** School of Information Science and Technology, Northwest University, Xi’an 710127, China; zhang_wei@nwu.edu.cn (W.Z.); lib@nwu.edu.cn (B.L.); jwang@nwu.edu.cn (J.W.); pjyxida@nwu.edu.cn (J.P.)

**Keywords:** directional modulation, physical layer security, multiple antenna arrays, artificial noise, robustness

## Abstract

Directional modulation (DM) technique has the ability to enhance the physical layer security (PLS) of wireless communications. Conventional DM schemes are usually based on a single antenna array with the basic assumption that eavesdroppers (Eves) and legitimate users (LUs) are in different directions. However, it is possible that Eves are in the same direction as LUs in practical applications. As a result, signals received by Eves will be approximately the same or even in better quality than those received by LUs. To address the neighbor security issue, we introduce a multiple antenna arrays model at the transmitter side with the help of the artificial noise (AN)-aided DM technique to achieve secure and precise DM transmission in this paper. Meanwhile, to recover the mixed useful signals, two novel DM schemes based on single- and multi-carrier multiple antenna arrays model are proposed, respectively. In addition, the symbol error rate (SER), secrecy rate, and robustness performance of the proposed DM schemes were analyzed and simulated. Simulations validate the effectiveness of the proposed DM schemes and demonstrate that multiple antenna arrays model based DM methods outperform single antenna array model aided DM methods in security.

## 1. Introduction

Information security has always been an important problem for wireless communications, which provide a wide coverage at the cost of exposing information to undesired users with eavesdroppers (Eves) amongst them [1]. Traditionally, the higher-layer encryption techniques have been widely adopted to handle the security problem. However, with the explosive development of mobile Internet, the key-based methods need a secure channel to exchange the private key, which may be insufficient or even not suitable [2,3]. Fortunately, physical layer security (PLS) exploiting the characteristics of wireless channels, e.g., interferences, noise, and fading, enables the secure communications without extra resources and signaling overhead, which can be considered as a significant complement to traditional cryptographic techniques [4,5].

Directional modulation (DM), as an emerging promising physical layer wireless security technique, has attracted widespread concern and attention from many researchers globally over the past decade [6]. The initial concept of DM can be traced back to the near-field direct antenna modulation (NFDAM) techniques in [7,8], where direction-dependent signals are formed by a transmit beam and a reflected beam at the radio frequency (RF) frontend. An angle-dependent DM technique for phased arrays was proposed in [9]. By changing the phase shift of each element, a symbol with desired amplitude and phase for generating a standard constellation can be projected along a pre-specified direction, while purposely scrambling the constellation to be very hard to demodulate in other unintended directions. In [10], the authors further demonstrated a DM transmitter to verify the feasibility of the proposed DM technique. A dual-beam antenna array excited by the I and Q signals was put forward to perform secure wireless DM transmission in [11]. Pattern synthesis approach was applied to DM systems in [12,13]. This technique has been further extended to antenna subset modulation (ASM) technique in [14] for PLS communications. Bit error rate (BER) driven DM synthesis approaches were demonstrated in [9,10,15] to secure wireless communications. A switched antenna array [16], a linear sparse array [17], a sparse linear array [18], and a 4-D antenna array [19] were proposed to perform wireless DM transmission at the RF frontend. Afterwards, the artificial noise (AN) and DM concepts were first linked in [20], and further extended via the orthogonal vector approach in [21], which transferred the DM synthesis method from the RF frontend to the baseband. Furthermore, the orthogonal vector approach for an angle-dependent multi-beam DM system was implemented in [22]. Then, robust synthesis methods based on AN for secure single-beam and multi-beam DM were demonstrated by Hu et al. [23] and Shu et al. [24], respectively. The AN was also employed in [25,26,27] to secure DM transmission using the zero-forcing, precoding vector, and iteration convex optimization methods, respectively.

However, the above-mentioned single- and multi-beam DM schemes can only achieve angle-dependent secure transmission, the security of which cannot be guaranteed once an Eve is aligned with the desired direction or very close to the desired user. To solve this neighbor security problem, one solution is to adopt a frequency diverse array (FDA) to synthesize both angle and range dependent DM [28,29,30,31]; another solution is to utilize a multi-path model, where signals via both line of sight (LOS) and reflected paths are combined at the receiver side [32,33,34,35,36,37,38,39]. The above solutions are all based on a single antenna array model. There are few studies regarding secure DM transmission for a multiple antenna arrays model. In [40], the authors presented a concept of secure and precise wireless transmission by using DM, random subcarrier selection with randomization procedure, and phase alignment at the DM transmitter for PLS. Inspired by the multiple antenna arrays idea presented in [40,41,42], we are dedicated to achieving secure and precise DM transmissions based on a multiple antenna arrays model for the purpose of addressing the neighbor security issue that exists in traditional DM schemes. This is the main motivation and goal of our paper.

Overall, the main novelties and contributions of our work can be summarized as follows.

(1) A multiple antenna arrays model combined with AN technique is introduced into DM designs to settle the neighbor security problem in traditional single antenna array based DM schemes for PLS enhancement.

(2) Due to the introduction of the multiple antenna arrays model, the useful signals are mixed together at the receiver side. To recover the intact useful signals, two secure and precise DM transmission schemes based on single- and multi-carrier multiple antenna arrays model are proposed, respectively. For each scheme, the architectures of the transmitter and the legitimate receiver are elaborately designed, individually. In single-carrier DM scheme, the receive multi-beamforming method is adopted at the receiver side. In multi-carrier DM scheme, the multi-carrier method is utilized at the transmitter side, and the mixed useful signals can be easily recovered by using multiple band-pass filters at the receiver side.

(3) Three important metrics for the proposed DM schemes, namely the symbol error rate (SER), secrecy rate, and robustness, were deduced and verified with simulations. The comparisons among the proposed DM schemes and the traditional AN-aided single antenna array DM schemes were also performed.

The proposed schemes can be typically applied to the future LOS communication environments. Their major application scenarios include secure military communications, satellite communications, unmanned aerial vehicle (UAV) networks, Internet of Things (IoT), and millimeter communications [43].

The remaining part of the paper is organized as follows. The principle of multiple antenna arrays based DM transmission is briefly introduced in Section 2. Section 3 and Section 4 illustrate the principles of the proposed DM schemes based on single-carrier multiple antenna arrays model and multi-carrier multiple antenna arrays model, respectively. Section 5 presents the SER, secrecy rate, and the robustness performance of the proposed DM schemes, and further compares the proposed DM schemes with the traditional AN-aided single antenna array DM schemes. Then, performance simulations and discussions are shown in Section 6. Section 7 concludes this paper and points out the future research directions.

Throughout this paper, boldface uppercase, boldface lowercase, and lowercase letters are used for matrices, vectors, and scalars, respectively. The superscripts ${\left(\cdot \right)}^{*}$, ${\left(\cdot \right)}^{T}$, ${\left(\cdot \right)}^{H}$, and ${\left(\cdot \right)}^{-1}$ denote complex conjugate, transpose, complex conjugate transpose, and inverse operators, respectively. The function card(·) represents the cardinality of a set. The operator E(·) means the statistical expectation. The sign $\left|\cdot \right|$ denotes the absolute value of a scalar. The notation ${\left[\cdot \right]}^{†}$ refers to max{0,·}. 0 is the all zero vector, R is the set of all real numbers, and C is the set of all complex numbers. $I_{N}$ represents an N×N identity matrix. The expression $x∼CN(0,\sigma^{2})$ means that random variable *x* obeys a circularly symmetric complex Gaussian (normal) distribution with zero mean and variance $\sigma^{2}$. The abbreviations “Tx”, “mc”, “sc”, “sa” in the figures of this paper refer to the symbol stream transmitted by transmitter, the multi-carrier multiple antenna arrays DM scheme, the single-carrier multiple antenna arrays DM scheme, and the single antenna array DM scheme, respectively.

## 2. Principle of Multiple Antenna Arrays based DM Transmission

### 2.1. System Model

**Figure 1:**
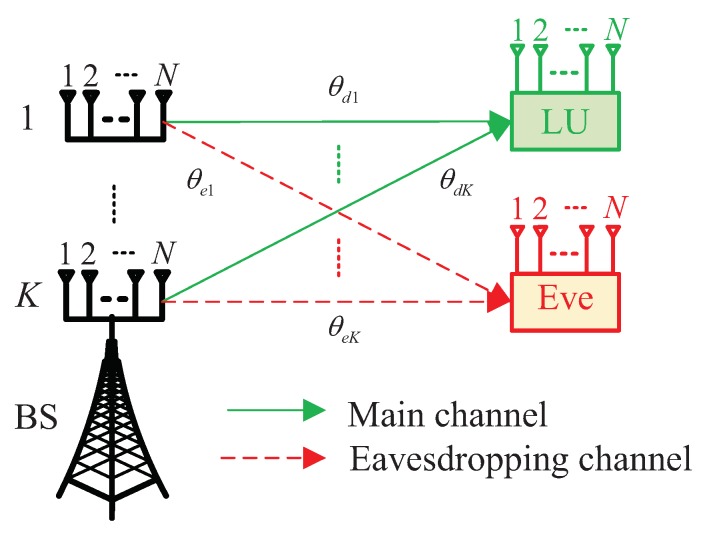
System model.

In this paper, we take a multiple-input multiple-output (MIMO) downlink system into account, as depicted in Figure 1. The system mainly comprises a base station (BS) with *K* (K≥2) non-collocated transmit antenna arrays, each of which is an *N*-element isotropic uniform linear array (ULA) with an interelement spacing of *d*, one LU with multiple receive antennas, and one passive Eve wishing to intercept the confidential information sent from the BS to the LU.

For the sake of analysis, path loss is normalized and neglected. The direction angle between the *k*th (k=1,2,⋯,K) transmit antenna array and the LU is denoted by $\theta_{dk}$. Following Hu et al. [23] and Shu et al. [24], the normalized steering vector for the *k*th transmit antenna array can be calculated by

(1) $$\begin{array}{c} h\left(\theta_{dk}\right)=\frac{1}{\sqrt{N}}{\left[\underset{h_{1}\left(\theta_{dk}\right)}{\underset{︸}{e^{j2\pi \varphi_{\theta_{dk}}\left(1\right)}}},\cdots ,\underset{h_{n}\left(\theta_{dk}\right)}{\underset{︸}{e^{j2\pi \varphi_{\theta_{dk}}\left(n\right)}}},\cdots ,\underset{h_{N}\left(\theta_{dk}\right)}{\underset{︸}{e^{j2\pi \varphi_{\theta_{dk}}\left(N\right)}}}\right]}^{H}, \end{array}$$

where $\varphi_{\theta_{dk}}\left(n\right)$ is given by

(2) $$\begin{array}{c} \varphi_{\theta_{dk}}\left(n\right)=\frac{\left(n-1\right)d\cos\theta_{dk}}{\lambda_{k}}, \end{array}$$

where $\lambda_{k}$ is the carrier wavelength at the frequency $f_{ck}$ of interest for the *k*th transmit antenna array.

Likewise, the direction angle between the *k*th (k=1,2,⋯,K) transmit antenna array and the Eve is denoted by $\theta_{ek}$.

### 2.2. The Principle of Secure and Precise DM Transmission

**Figure 2:**
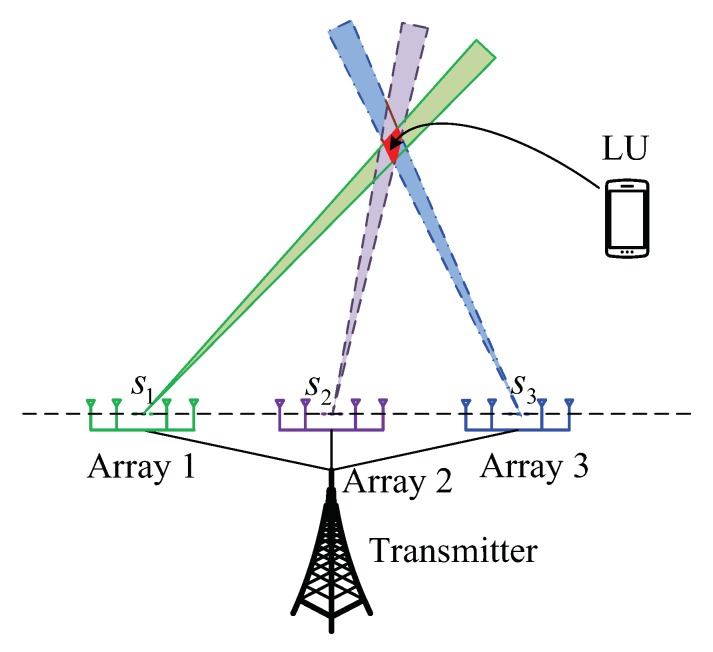
The principle of secure and precise wireless transmission for three antenna arrays with the help of AN-aided DM technique.

Without loss of generality, three antenna arrays at the transmitter side are taking as an example to elaborate the principle of secure and precise DM transmission, as shown in Figure 2.

Assume that three antenna arrays are utilized to transmit symbol streams $s_{1}$, $s_{2}$, and $s_{3}$ with AN-aided DM technique simultaneously. The green (solid line), purple (dashed line), and blue (dot dash line) areas represent the main information beam of the three antenna arrays, respectively.

As we know, in traditional AN-aided DM schemes using a single antenna array model, only signals in the main beam region can be demodulated perfectly, while signals outside the main beam region will be seriously polluted by inserted AN.

In our multiple antenna arrays based DM schemes, at first, we consider the symbol stream transmitted by the first antenna array. Obviously, the radiation region of the first antenna array can be divided into three parts. The first is the region outside the main beam region. The second is the main beam except the controlled region for the LU, as shown in Figure 2. The last is the controlled region for the LU, which is the overlap of main beams of the three transmit antenna arrays. Different from a single antenna array based AN-aided DM schemes, in our proposed DM schemes, signals in the previous two parts are both seriously polluted by the inserted AN and the AWGN (additive white Gaussian noise), while observations for the LU are a simple summation of useful signals and the AWGN. Thus, the LU can easily recover the useful signals transmitted from the first antenna array. Likewise, the symbol streams transmitted by the second and the third antenna array except the controlled area for the LU are all seriously polluted by the inserted AN. Therefore, we can employ multiple antenna arrays to transmit useful signals to a specified small neighborhood without any pollution. That is the principle to achieve secure and precise wireless transmission using a multiple antenna arrays model with the help of AN-aided DM technique.

Owing to the introduction of the multiple antenna arrays model, the useful signals are mixed together at the receiver side. Therefore, two secure and precise DM transmission schemes based on a single-carrier multiple antenna arrays model in Section 3 and a multi-carrier multiple antenna arrays model in Section 4 are proposed to recover the mixed useful signals, respectively.

## 3. Secure and Precise DM Scheme Based on A Single-Carrier Multiple Antenna Arrays Model

In this section, a case where the BS is trying to transmit confidential messages to the LU at a predetermined position using a single-carrier multiple antenna arrays model is considered. The BS is assumed to be able to communicate with the LU, and the direction angles between *K* transmit antenna arrays and the LU are known. Moreover, we assume there only exists a passive eavesdropper.

### 3.1. The Architecture of BS Based on a Single-Carrier Multiple Antenna Arrays Model

**Figure 3:**
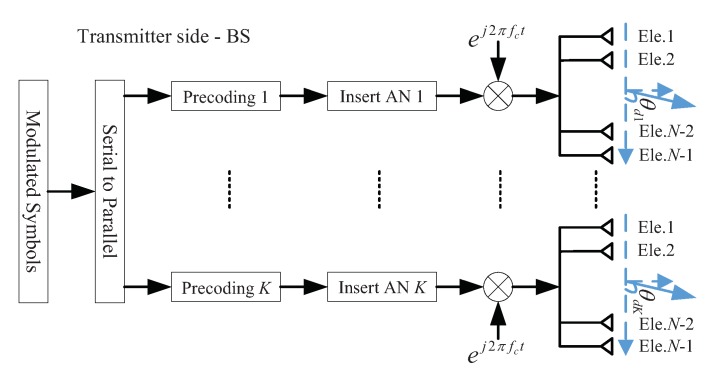
The architecture of the BS for the proposed DM scheme based on a single-carrier multiple antenna arrays model.

The architecture of the BS is shown in Figure 3, which employs *K* ULAs with all antenna arrays emitting the same carrier to steer the *K* confidential information beams towards the LU simultaneously.

Unlike traditional beamforming, the AN-aided beamforming technique has been adopted extensively under the background of secure wireless communications due to its perfect security performance. The received information in the desired direction will not be affected by AN, while the messages in other directions will be seriously interfered, which is beneficial for PLS enhancement. Therefore, AN-aided beamforming technique is introduced to achieve DM in the proposed scheme to achieve secure and precise wireless transmission.

Let $s=[s_{1},s_{2},\cdots ,s_{K}]$ denote the symbol vector formed by combining the *K* pre-transmitting baseband symbols via multiple antenna arrays. From another perspective, s can also be regarded as a *K*-element symbol frame. The *k*th symbol $s_{k}$ is valued from the set $B=\{b_{1},b_{2},\cdots ,b_{M}\}$ with card(B)=M, which comprises all symbols belonging to an *M*-ary amplitude and phase modulation, such as PSK (phase shift keying) or QAM (quadrature amplitude modulation), i.e., $s_{k}\in B$. Suppose each baseband symbol is normalized, i.e., satisfies $E[|s_{k}^{*}s_{k}|]=1$.

At first, do serial-to-parallel conversion for each symbol vector s to match *K* transmit antenna arrays.

Next, before transmitting, the *k*th symbol $s_{k}$ should be processed by AN-aided beamforming to match the *N* elements of the *k*th transmit antenna array. The transmitted signal vector $u_{k}={\left[u_{1k},u_{2k},\cdots ,u_{Nk}\right]}^{T}$ for the *N* antenna elements can be given by

(3) $$\begin{array}{c} u_{k}=\beta_{1}\sqrt{P_{s}}v_{k}s_{k}+\beta_{2}\sqrt{P_{s}}T_{k}z_{k}, \end{array}$$

where $P_{s}$ is the total transmit power for each symbol; $z_{k}$ is an *N*-tuple vector consisting of independent and identically distributed (i.i.d.) complex Gaussian random variables with the probability density function (PDF) being $CN(0_{N\times 1},I_{N})$, i.e., the inserted AN for the *k*th antenna array; $\beta_{1}\in \left(0,1\right]$ and $\beta_{2}\in \left[0,1\right)$ are information and AN power allocation factors, respectively, satisfying $\beta_{1}^{2}+\beta_{2}^{2}=1$; $v_{k}\in C{}^{N\times 1}$ is the transmit beamforming vector, which projects the useful signal $s_{k}$ to the desired direction; and $T_{k}\in C{}^{N\times N}$ is the projection matrix for controlling the direction of AN.

Then, our primary task is to find the transmit beamforming vector $v_{k}$ and the AN projection matrix $T_{k}$ for the *k*th transmit antenna array. Here, the AN projection matrix $T_{k}$ enforces the AN into null-space of the desired steering vector or the eavesdropper steering vector with a slight or no leakage of AN power to the desired steering space, while the precoding vector for the useful information is to convey the major power of useful information to the desired steering space with a small fraction or no leakage to its null-space.

Different from traditional beamforming, here, DM signals are synthesized in the baseband. The excitation signal vector of the useful signal corresponds to the steering vector of the desired direction. Therefore, the transmit beamforming vector $v_{k}$ can be designed as

(4) $$\begin{array}{c} v_{k}=h\left(\theta_{dk}\right). \end{array}$$

Obviously, the transmit beamforming vector $v_{k}$ has the normalization characteristic, i.e.,

(5) $$\begin{array}{c} h^{H}\left(\theta_{dk}\right)v_{k}=1. \end{array}$$

To prevent the inserted AN to interfere the confidential information $s_{k}$, we project the AN onto the null space of the steering vector $h(\theta_{dk})$ for the desired direction. Therefore, the projection matrix $T_{k}$ can be devised as

(6) $$\begin{array}{c} T_{k}=I_{N}-h\left(\theta_{dk}\right)h^{H}\left(\theta_{dk}\right). \end{array}$$

Apparently, the projection matrix $T_{k}$ has the orthogonal property, i.e.,

(7) $$\begin{array}{cc} h^{H}\left(\theta_{dk}\right)T_{k} & =h^{H}\left(\theta_{dk}\right)\left\{I_{N}-h\left(\theta_{dk}\right)h\left(\theta_{dk}\right)\right\}\\ \; & =h^{H}\left(\theta_{dk}\right)-h^{H}\left(\theta_{dk}\right)\\ \; & =0. \end{array}$$

After up-converting to RF using the same carrier $f_{c}$ for all transmit antenna arrays, the radiating signal $x_{k}={\left[x_{1k},x_{2k},\cdots ,x_{Nk}\right]}^{T}$ for the *k*th *N*-element antenna array at time *t* can be given by

(8) $$\begin{array}{c} x_{k}\left(t\right)=u_{k}\left(t\right)\cdot e^{j2\pi f_{c}t}. \end{array}$$

Finally, all radiating signals for each symbol vector s can compose a transmitted signal vector, i.e.,

(9) $$\begin{array}{c} x\left(t\right)=\sum_{k=1}^{K}x_{k}\left(t\right)=e^{j2\pi f_{c}t}\cdot \sum_{k=1}^{K}\left[\beta_{1}\sqrt{P_{s}}h\left(\theta_{dk}\right)s_{k}+\beta_{2}\sqrt{P_{s}}T_{k}z_{k}\right]. \end{array}$$

It is noted that the BS transmits every symbol of s to the LU from different directions with the same carrier simultaneously. Evidently, only the LU can receive all the useful signals, while the Eve cannot receive any useful information at all.

### 3.2. The Architecture of the LU Based on a Single-Carrier Multiple Antenna Arrays Model

**Figure 4:**
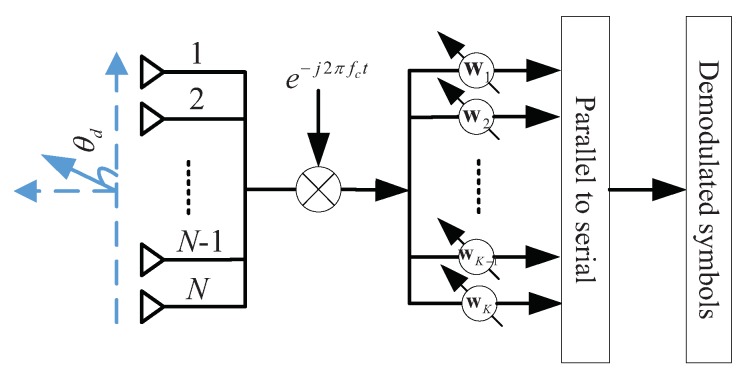
The architecture of the LU for the proposed DM based on a single-carrier multiple antenna arrays.

The architecture of the LU based on a single-carrier multiple antenna arrays model is demonstrated in Figure 4. To fairly compare the proposed DM technique and other DM techniques, without loss of generality, the normalized narrow-band LOS AWGN channel with perfect symbol-rate sampling and synchronization in free space is assumed throughout the paper.

After down-converting to baseband using the same carrier $f_{c}$, the received signal $y^{\prime }={\left[y_{1}^{\prime },\cdots ,y_{N}^{\prime }\right]}^{T}$ of the LU at time *t* can be written as

(10) $$\begin{array}{c} y^{\prime }\left(t\right)=e^{-j2\pi f_{c}t}\sum_{k=1}^{K}x_{k}\left(t\right)+n^{\prime }, \end{array}$$

where $n^{\prime }={\left[n_{1}^{\prime },\cdots ,n_{N}^{\prime }\right]}^{T}∼CN\left(0_{N\times 1},\sigma_{n^{\prime }}^{2}I_{N}\right)$ is the complex AWGN vector.

Substituting Equations (3), (4) and (8) into Equation (10), the received signal $y^{\prime }$ can be further simplified as

(11) $$\begin{array}{c} y^{\prime }=\sum_{k=1}^{K}\left(\beta_{1}\sqrt{P_{s}}h\left(\theta_{dk}\right)s_{k}+\beta_{2}\sqrt{P_{s}}T_{k}z_{k}\right)+n^{\prime }. \end{array}$$

As we can see from Equation (11), the *K* symbols in each symbol frame are mixed together at the receiver side. We have to design a receive multi-beamforming scheme to recover the *K* symbols. Each set of weight coefficients corresponds to a receive beam for the recovery of one symbol.

For example, to obtain the *l*th (l∈{1,2,⋯,K}) symbol $s_{l}$ from the direction $\theta_{dl}$, it is necessary to steer the array nulls precisely to the other K−1 directions. The subspace of the K−1 interference directions is given by

(12) $$\begin{array}{c} A=[h\left(\theta_{d1}\right),\cdots ,h\left(\theta_{d(l-1)}\right),h\left(\theta_{d(l+1)}\right),\cdots ,h\left(\theta_{dK}\right)]. \end{array}$$

The orthogonal complement space of A is denoted by $P_{A}^{⊥}$, which is given by

(13) $$\begin{array}{c} P_{A}^{⊥}=I_{N}-A{\left(A^{H}A\right)}^{-1}A^{H}. \end{array}$$

Obviously, $P_{A}^{⊥}$ is a Hermitian matrix. We have

(14) $$\begin{array}{c} P_{A}^{⊥}={\left(P_{A}^{⊥}\right)}^{H}. \end{array}$$

Therefore, the weight vector of the *l*th receive beam can be designed as

(15) $$\begin{array}{c} w_{l}=P_{A}^{⊥}h\left(\theta_{dl}\right). \end{array}$$

As is well known from matrix theory, if subspace A is orthogonal to subspace B, then the vector projected in subspace A is also perpendicular to subspace B. Hence, arbitrary vector $h(\theta_{dk})$, k={1,⋯,l−1,l+1,⋯,K} will satisfy that

(16) $$\begin{array}{c} w_{l}^{H}h\left(\theta_{dk}\right)=0. \end{array}$$

In other words, the beam formed by the weight vector $w_{l}$ can result in null steering in other K−1 desired directions.

Let $\xi =h^{H}\left(\theta_{dl}\right)A{\left(A^{H}A\right)}^{-1}A^{H}h\left(\theta_{dl}\right)$ and $n_{l}=w_{l}^{H}n^{\prime }$. Therefore, the *l*th (l∈{1,2,⋯,K}) received symbol $y_{l}$ after the receive multi-beamforming processing can be expressed as

(17) $$\begin{array}{c} y_{l}=w_{l}^{H}y^{\prime }=s_{l}\beta_{1}\sqrt{P_{s}}\left(1-\xi \right)+n_{l}, \end{array}$$

where ξ is a tiny real-valued scaling factor and $n_{l}$ is the AWGN with zero mean and variance $\sigma_{n_{l}}^{2}$.

**Proof.**  Please refer to Appendix A. □

It is noted that the received symbol $y_{l}$ is a simple summation of the useful signal $s_{l}$ and AWGN $n_{l}$. From Equation (17), the LU can easily recover the *l*th (l∈{1,2,⋯,K}) symbol transmitted from the BS.

Finally, we can represent the *K* received symbols as a received vector, i.e., a received symbol frame,

(18) $$\begin{array}{c} y={\left[y_{1},\cdots ,y_{K}\right]}^{T}={\left[w_{1},\cdots ,w_{K}\right]}^{H}y^{\prime }. \end{array}$$

However, for the Eve, we consider two scenarios. One case is that the Eve is not in the same direction as the LU for any antenna arrays. The other worse case is that the Eve is in the same direction as the desired receiver for one antenna array and not in the same direction for another antenna array.

In Case 1, the assumption condition is denoted as $\theta_{dl}\neq \theta_{ek}$ for arbitrary l,k∈{1,2,⋯,K}. The received message can be written as Equation (19), where $n_{l}^{E}=h^{H}\left(\theta_{el}\right)n^{\prime }∼CN\left(0,\sigma_{n_{l}^{E}}^{2}\right)$ is the AWGN with zero mean and variance $\sigma_{n_{l}^{E}}^{2}$.

(19) $$\begin{array}{cc} y_{l} & =h^{H}\left(\theta_{el}\right)y^{\prime }\\ \; & =h^{H}\left(\theta_{el}\right)\left[\sum_{k=1}^{K}\left(\beta_{1}\sqrt{P_{s}}h\left(\theta_{dk}\right)s_{k}+\beta_{2}\sqrt{P_{s}}T_{k}z_{k}\right)+n^{\prime }\right]\\ \; & =\underset{\mathrm{Scrambled}\;\mathrm{signal}}{\underset{︸}{\beta_{1}\sqrt{P_{s}}h^{H}\left(\theta_{el}\right)h\left(\theta_{dl}\right)s_{l}}}+\underset{\mathrm{Equivalent}\;\mathrm{AN}}{\underset{︸}{\beta_{1}\sqrt{P_{s}}\sum_{k=1,k\neq l}^{K}h^{H}\left(\theta_{el}\right)h\left(\theta_{dk}\right)s_{k}}}+\underset{\mathrm{AN}}{\underset{︸}{\beta_{2}\sqrt{P_{s}}\sum_{k=1}^{K}h^{H}\left(\theta_{el}\right)T_{k}z_{k}}}+\underset{\mathrm{AWGN}}{\underset{︸}{n_{l}^{E}}}. \end{array}$$

It can be seen from Equation (19) that the received signal is made up of four components. The first is the scrambled signal. When $\theta_{dl}\neq \theta_{ek}$, we have $h^{H}\left(\theta_{el}\right)h\left(\theta_{dl}\right)=\delta \cdot e^{j\Delta \varphi }$. It is obvious that there exists an amplitude difference $\delta \beta_{1}\sqrt{P_{s}}$ and a phase difference Δφ between the received symbol and the original signal $s_{l}$ for the first part, which can disrupt the standard constellation format. The second component is the equivalent AN caused by the other K−1 symbols in each symbol frame, which results in a high interference level for the recovery of symbol $s_{l}$. The third part is the superimposed AN from the *K* transmit antenna arrays. The fourth component is the AWGN. Therefore, the Eve can hardly recover the confidential information from the seriously interfered signals, which indicates that the transmission security can be guaranteed in the first scenario.

In Case 2, a worse case, the assumption condition can be described as $\theta_{el}=\theta_{dl}$, for a fixed *l*, (l∈{1,2,⋯,K}), and $\theta_{el}\neq \theta_{dk}$, for arbitrary *k*, k∈{1,⋯,l−1,l+1,⋯,K}. Then, the received signal from the *l*th (l∈{1,2,⋯,K}) transmit antenna array can be expressed as Equation (20).

(20) $$\begin{array}{cc} y_{l} & =h^{H}\left(\theta_{el}\right)y^{\prime }\\ \; & =\underset{\mathrm{Information}}{\underset{︸}{\beta_{1}\sqrt{P_{s}}s_{k}}}+\underset{\mathrm{Equivalent}\;\mathrm{AN}}{\underset{︸}{\beta_{1}\sqrt{P_{s}}\sum_{k=1,k\neq l}^{K}h^{H}\left(\theta_{dl}\right)h\left(\theta_{dk}\right)s_{k}}}+\underset{\mathrm{AN}}{\underset{︸}{\beta_{2}\sqrt{P_{s}}\sum_{k=1,k\neq l}^{K}h^{H}\left(\theta_{dl}\right)T_{k}z_{k}}}+\underset{\mathrm{AWGN}}{\underset{︸}{n_{l}}}. \end{array}$$

Likewise, the other received signal from the *k*th (k∈{1,⋯,l−1,l+1,⋯,K}) transmit antenna array can be expressed as Equation (21), where $n_{k}=h^{H}\left(\theta_{ek}\right)n^{\prime }$ is the AWGN with PDF being $CN(0,\sigma_{n_{k}^{E}}^{2})$.

(21) $$\begin{array}{cc} y_{k} & =h^{H}\left(\theta_{ek}\right)y^{\prime }\\ \; & =h^{H}\left(\theta_{ek}\right)[\sum_{m=1}^{K}\left(\beta_{1}\sqrt{P_{s}}h\left(\theta_{dm}\right)s_{m}+\beta_{2}\sqrt{P_{s}}T_{m}z_{m}\right)+n^{\prime }]\\ \; & =\underset{\mathrm{Scrambled}\;\mathrm{information}}{\underset{︸}{\beta_{1}\sqrt{P_{s}}h^{H}\left(\theta_{ek}\right)h\left(\theta_{dk}\right)s_{k}}}+\underset{\mathrm{Equivalent}\;\mathrm{AN}}{\underset{︸}{\beta_{1}\sqrt{P_{s}}\sum_{m=1,m\neq k}^{K}h^{H}\left(\theta_{ek}\right)h\left(\theta_{dm}\right)s_{m}}}+\underset{\mathrm{AN}}{\underset{︸}{\beta_{2}\sqrt{P_{s}}\sum_{m=1}^{K}h^{H}\left(\theta_{ek}\right)T_{m}z_{m}}}+\underset{\mathrm{AWGN}}{\underset{︸}{n_{k}}}, \end{array}$$

It can also be observed from Equation (20) that the received signal from the *l*th (l∈{1,2,⋯,K}) antenna array is severely contaminated by the other K−1 data streams, K−1 inserted AN and the AWGN. Similarly, from Equation (21), we can find that the received signal from the *k*th (k∈{1,⋯,l−1,l+1,⋯,K}) antenna array is also scrambled by the term $h^{H}\left(\theta_{ek}\right)h\left(\theta_{dk}\right)$, contaminated by the equivalent AN, inserted AN, and the AWGN. Therefore, even if the Eve is aligned with or very close to one transmit direction, the transmission security can also be ensured owing to *K* symbols emitting simultaneously from different directions, which outperforms traditional AN-aided DM schemes using a single antenna array.

## 4. Secure and Precise DM Scheme Based on A Multi-Carrier Multiple Antenna Arrays Model

In this section, a scenario where the BS intends to send the confidential information to the LU at a fixed position using a multi-carrier multiple antenna arrays model is considered. The assumptions are the same as the case in Section 3. In addition, there exists only a passive Eve.

### 4.1. The Architecture of the BS Based on a Multi-Carrier Multiple Antenna Arrays Model

**Figure 5:**
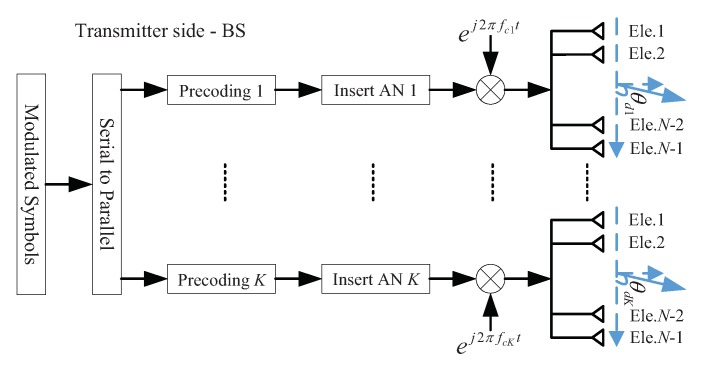
The architecture of the BS for the proposed DM based on a multi-carrier multiple antenna arrays model.

The architecture of the BS based on a multi-carrier multiple antenna arrays model is shown in Figure 5. Compared with the architecture in Section 3.1, the difference is that *K* transmit antenna arrays emitting different carriers. For the *k*th transmit antenna array, the carrier frequency can be expressed as ($f_{ck}$ (k=1,2,⋯,K)).

Before up-converting to RF, the modulated symbols are processed with the same manner in Section 3.1. Therefore, for the *k*th transmitted symbol, we still have

(22) $$\begin{array}{c} u_{k}=\beta_{1}\sqrt{P_{s}}h\left(\theta_{dk}\right)s_{k}+\beta_{2}\sqrt{P_{s}}\left(I_{N}-h\left(\theta_{dk}\right)h^{H}\left(\theta_{dk}\right)\right)z_{k}. \end{array}$$

Then, for the purpose of matching the *N* antenna elements for the *k*th transmit antenna array with carrier $f_{ck}$, the radiating signal $x_{k}={\left[x_{1k},x_{2k},\cdots ,x_{Nk}\right]}^{T}$ at time *t* can be obtained by

(23) $$\begin{array}{c} x_{k}\left(t\right)=u_{k}\left(t\right)\cdot e^{j2\pi f_{ck}t}. \end{array}$$

Eventually, all *K* radiating signal vectors for each symbol frame s can be expressed as

(24) $$\begin{array}{c} x\left(t\right)=\sum_{k=1}^{K}x_{k}\left(t\right)=\sum_{k=1}^{K}\left[u_{k}\left(t\right)\cdot e^{j2\pi f_{ck}t}\right]. \end{array}$$

It is also noted that the BS sends each symbol of s towards the LU from distinct directions with different carriers concurrently. Obviously, just the LU can receive the complete information without contamination. Meanwhile, due to the introduction of AN, the Eve can hardly intercept any useful signals seriously interfered by AN.

### 4.2. The Architecture of the LU Based on a Multi-Carrier Multiple Antenna Arrays Model

**Figure 6:**
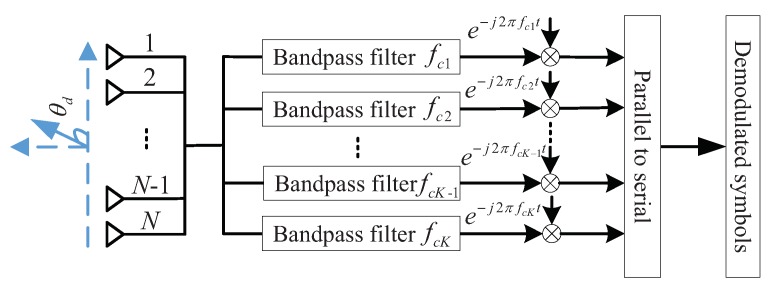
The architecture of the LU for the proposed DM based on a multi-carrier multiple antenna arrays model.

To match the architecture of the BS, the architecture of the LU based on a multi-carrier multiple antenna arrays model is redesigned in Figure 6. In practical applications, the receive antenna array for the proposed DM scheme can be replaced by a single antenna to further simplify the DM system.

After passing through the LOS channel in free space, all symbols of each symbol frame with different carriers are mixed together at the receiver side. For the recovery of the *k*th (k=1,…,K) symbol, at first, the mixed signals should be processed with a band-pass filter with a specified center frequency $f_{ck}$ and an appointed bandwidth BW.

Without loss of generality, a narrow-band channel with perfect symbol synchronization and symbol-rate sampling is assumed. Then, after the down conversion, the *k*th received baseband signal at time *t* can be expressed as

(25) $$\begin{array}{c} y_{k}^{\prime }\left(t\right)=h^{H}\left(\theta_{dk}\right)x_{k}\left(t\right)\cdot e^{-j2\pi f_{ck}t}+n_{k}^{\prime }\left(t\right), \end{array}$$

where $n_{k}^{\prime }\left(t\right)$ is the complex AWGN between the *k*th transmitter and the LU with the distribution being $n_{k}^{\prime }\left(t\right)∼CN\left(0,\sigma_{k}^{\prime 2}\right)$.

Substituting Equations (22)–(24) into Equation (25), and digitizing the *k*th received signal during the symbol period, we have

(26) $$\begin{array}{cc} y_{k} & =h^{H}\left(\theta_{dk}\right)u_{k}+n_{k}\\ \; & =\beta_{1}\sqrt{P_{s}}s_{k}+n_{k}, \end{array}$$

where the random variable $n_{k}$ denotes the AWGN for the *k*th received symbol, which is i.i.d. with $n_{k}∼CN\left(0,\sigma^{2}\right)$.

Obviously, each symbol is a simple summation of the useful signal and the AWGN. Therefore, the LU can easily recover the *k*th confidential symbol transmitted from the *k*th transmitter using Equation (26).

After receiving all of the transmitted symbols of an intact symbol frame during the symbol period, the received symbol frame can be written as

(27) $$\begin{array}{cc} y & =[y_{1},y_{2},\cdots ,y_{K}]\\ \; & =\beta_{1}\sqrt{P_{s}}\left[s_{1},s_{2},\cdots ,s_{K}\right]+n\\ \; & =\beta_{1}\sqrt{P_{s}}s+n, \end{array}$$

where $n=\left[n_{1},n_{2},\cdots ,n_{K}\right]∼CN\left(0_{1\times K},\sigma^{2}I_{K}\right)$ is the AWGN vector.

From Equation (27), each symbol frame transmitted from the BS can be easily recovered.

Similarly, for the Eve, the two scenarios in Section 3.2 are also considered in the following.

In Case 1, let the Eve be located at $\theta_{e}$, and $\theta_{e}\neq \theta_{dk}$, k∈{1,⋯,K}. Specifically, if the Eve is intended to eavesdrop the *l*th (l∈{1,⋯,K}) symbol, its received signal at time *t* can be given by

(28) $$\begin{array}{cc} y_{l}^{{}^{\prime }E}\left(t\right) & =h^{H}\left(\theta_{e}\right)x\left(t\right)\cdot e^{-j2\pi f_{cl}t}+n_{l}^{{}^{\prime }E}\left(t\right)\\ \; & =h^{H}\left(\theta_{dl}\right)\sum_{k=1}^{K}\left[u_{k}\left(t\right)\cdot e^{j2\pi f_{ck}t}\right]\cdot e^{-j2\pi f_{cl}t}+n_{l}^{{}^{\prime }E}\left(t\right)\\ \; & =h^{H}\left(\theta_{dl}\right)u_{l}\left(t\right)+h^{H}\left(\theta_{dl}\right)\sum_{k=1,k\neq l}^{K}\left[u_{k}\left(t\right)\cdot e^{j2\pi f_{ck}t}\right]\cdot e^{-j2\pi f_{cl}t}+n_{l}^{{}^{\prime }E}\left(t\right)\\ \; & =h^{H}\left(\theta_{dl}\right)\left[\beta_{1}\sqrt{P_{s}}h\left(\theta_{dl}\right)s_{l}+\beta_{2}\sqrt{P_{s}}T_{l}z_{l}\right]+h^{H}\left(\theta_{dl}\right)\sum_{k=1,k\neq l}^{K}\left[u_{k}\left(t\right)\cdot e^{j2\pi f_{ck}t}\right]\cdot e^{-j2\pi f_{cl}t}+n_{l}^{{}^{\prime }E}\left(t\right)\\ \; & =\underset{\mathrm{useful}\;\mathrm{Signal}}{\underset{︸}{\beta_{1}\sqrt{P_{s}}s_{l}}}+\underset{\mathrm{Equivalent}\;\mathrm{Mixed}\;\mathrm{Noise}}{\underset{︸}{h^{H}\left(\theta_{dl}\right)\sum_{k=1,k\neq l}^{K}\left[u_{k}\left(t\right)\cdot e^{j2\pi f_{ck}t}\right]\cdot e^{-j2\pi f_{cl}t}}}+\underset{\mathrm{AWGN}}{\underset{︸}{n_{l}^{{}^{\prime }E}\left(t\right)}}. \end{array}$$

where $n_{l}^{{}^{\prime }E}\left(t\right)$ is the complex AWGN with the distribution $n_{l}^{{}^{\prime }E}\left(t\right)∼CN\left(0,\sigma_{e}^{2}\right)$.

From Equation (28), the *l*th received signal of the Eve is composed of four components. The first is the *l*th distorted signal, the second is the inserted AN, the third is the equivalent mixed noise from the other transmitters, and the last is the AWGN.

Furthermore, in Case 2, let the Eve be located at $\theta_{e}$, and $\theta_{e}=\theta_{dl}$, l∈{1,⋯,K}. Specifically, when the Eve is meant to eavesdrop the *l*th symbol, the received signal at time *t* in Equation (28) can be further reorganized as

(29) $$\begin{array}{cc} y_{l}^{{}^{\prime }E}\left(t\right) & =h^{H}\left(\theta_{e}\right)x\left(t\right)\cdot e^{-j2\pi f_{cl}t}+n_{l}^{{}^{\prime }E}\left(t\right)\\ \; & =h^{H}\left(\theta_{e}\right)\sum_{k=1}^{K}\left[u_{k}\left(t\right)\cdot e^{j2\pi f_{ck}t}\right]\cdot e^{-j2\pi f_{cl}t}+n_{l}^{{}^{\prime }E}\left(t\right)\\ \; & =h^{H}\left(\theta_{e}\right)u_{l}\left(t\right)+h^{H}\left(\theta_{e}\right)\sum_{k=1,k\neq l}^{K}\left[u_{k}\left(t\right)\cdot e^{j2\pi f_{ck}t}\right]\cdot e^{-j2\pi f_{cl}t}+n_{l}^{{}^{\prime }E}\left(t\right)\\ \; & =\underset{\mathrm{Distorted}\;\mathrm{Signal}}{\underset{︸}{h^{H}\left(\theta_{e}\right)h\left(\theta_{dl}\right)\beta_{1}\sqrt{P_{s}}s_{l}}}+\underset{\mathrm{AN}}{\underset{︸}{\beta_{2}\sqrt{P_{s}}h^{H}\left(\theta_{e}\right)T_{l}z_{l}}}+\underset{\mathrm{Equivalent}\;\mathrm{Mixed}\;\mathrm{Noise}}{\underset{︸}{h^{H}\left(\theta_{e}\right)\sum_{k=1,k\neq l}^{K}\left[u_{k}\left(t\right)\cdot e^{j2\pi f_{ck}t}\right]\cdot e^{-j2\pi f_{cl}t}}}+\underset{\mathrm{AWGN}}{\underset{︸}{n_{l}^{{}^{\prime }E}\left(t\right)}}. \end{array}$$

From Equation (29), it is observed that the *l*th symbol is interfered by the equivalent mixed noise and the AWGN. The Eve wants to intercept it requiring a higher signal-to-noise ratio (SNR). What is more, if the Eve is intended to eavesdrop the other K−1 symbols, e.g., the *m*th symbol (m∈{1,⋯,l−1,l+1,⋯,K}), the received signal in Equation (28) can be further converted into

(30) $$\begin{array}{cc} y_{m}^{{}^{\prime }E}\left(t\right) & =h^{H}\left(\theta_{e}\right)x\left(t\right)\cdot e^{-j2\pi f_{cm}t}+n_{m}^{{}^{\prime }E}\left(t\right)\\ \; & =h^{H}\left(\theta_{e}\right)\sum_{k=1}^{K}\left[u_{k}\left(t\right)\cdot e^{j2\pi f_{ck}t}\right]\cdot e^{-j2\pi f_{cm}t}+n_{m}^{{}^{\prime }E}\left(t\right)\\ \; & =h^{H}\left(\theta_{e}\right)u_{m}\left(t\right)+h^{H}\left(\theta_{dl}\right)u_{l}\left(t\right)e^{j2\pi (f_{cl}-f_{cm})t}+h^{H}\left(\theta_{e}\right)\sum_{k=1,k\neq l,k\neq m}^{K}\left[u_{k}\left(t\right)\cdot e^{j2\pi f_{ck}t}\right]\cdot e^{-j2\pi f_{cm}t}+n_{m}^{{}^{\prime }E}\left(t\right)\\ \; & =\underset{\mathrm{Distorted}\;\mathrm{Signal}}{\underset{︸}{\beta_{1}\sqrt{P_{s}}s_{m}h^{H}\left(\theta_{e}\right)h\left(\theta_{dm}\right)}}+\underset{\mathrm{AN}}{\underset{︸}{\beta_{2}\sqrt{P_{s}}h^{H}\left(\theta_{e}\right)T_{m}z_{m}}}+\underset{\mathrm{Equivalent}\;\mathrm{Noise}}{\underset{︸}{\beta_{1}\sqrt{P_{s}}s_{l}e^{j2\pi (f_{cl}-f_{cm})t}}}+\underset{\mathrm{Equivalent}\;\mathrm{Mixed}\;\mathrm{Noise}}{\underset{︸}{\sum_{k=1,k\neq l,m}^{K}\varepsilon_{k}}}+\underset{\mathrm{AWGN}}{\underset{︸}{n_{m}^{{}^{\prime }E}\left(t\right)}}. \end{array}$$

where $\varepsilon_{k}=\left[u_{k}\left(t\right)e^{j2\pi (f_{ck}-f_{cm})t}\right]h^{H}\left(\theta_{e}\right)$.

It can be observed from Equation (30) that each of the other K−1 received symbols includes five parts. The first is the distorted signal, the second is the injected AN, the third is equivalent noise caused by the *l*th rotated symbol, the fourth is the equivalent mixed noise introduced by the other K−2 symbols, and the last is the AWGN. Therefore, even if the Eve is aligned with or very close to one of the transmit directions, it still cannot recover the confidential information. Obviously, the transmission security is enhanced significantly owing to the use of multiple antenna arrays, which outperforms the traditional single antenna array based DM schemes.

## 5. Performance Analysis

The SER, secrecy rate, and robustness are three primary metrics for assessing the performance of DM systems [44]. Next, the SER, secrecy rate, and robustness of the proposed DM schemes are analyzed. Finally, the comparisons among the multiple antenna arrays DM schemes and the traditional single antenna array DM schemes are also provided.

### 5.1. SER

For the sake of simplicity, assume that all AWGNs are normalized and have the same distribution with zero mean and variance $\sigma^{2}$ for both LUs and Eves throughout the paper. Meanwhile, all the baseband symbols are normalized.

In the single-carrier case, using Equation (17), the SNR per symbol of the LU can be written as

(31) $$\begin{array}{c} r^{sc,LU}=\frac{P_{s}\beta_{1}^{2}{\left(1-\xi \right)}^{2}}{\sigma^{2}}. \end{array}$$

Likewise, in the multi-carrier case, by means of Equation (26), the SNR per symbol of the LU can be expressed as

(32) $$\begin{array}{c} r^{mc,LU}=\frac{P_{s}\beta_{1}^{2}}{\sigma^{2}}. \end{array}$$

By contrast, the SNR per symbol of the traditional single antenna array AN-aided DM schemes can be given by

(33) $$\begin{array}{c} r^{sa}=\frac{P_{s}\beta_{1}^{2}}{\sigma^{2}}. \end{array}$$

In this paper, quadrature phase-shift keying (QPSK) modulation is used by the proposed schemes. A closed-form expression for the SER of QPSK modulated signals in AWGN channel can be given by [45]

(34) Pe=1−(1−12erfcrr22)2=erfcrr22−14erfc2rr22,

where erfc(x)=22π·π·∫x∞exp(−t2)dt is the complementary error function of the standard normal distribution.

Meanwhile, the received signals for the Eve are scrambled by the eavesdropper steering vector.

At this time, for the single-carrier case, the SNR per symbol for the Eve can be transformed into

(35) $$\begin{array}{c} r^{sc,E}=\frac{P_{s}\beta_{1}^{2}h^{H}\left(\theta_{el}\right)h\left(\theta_{dl}\right)h^{H}\left(\theta_{dl}\right)h\left(\theta_{el}\right)}{\sigma^{2}}. \end{array}$$

Likewise, the SNR per symbol for the Eve in the multi-carrier case can be rewritten as

(36) $$\begin{array}{c} r^{mc,E}=\frac{P_{s}\beta_{1}^{2}|h^{H}\left(\theta_{e}\right)h\left(\theta_{dl}\right){|}^{2}}{\sigma^{2}}. \end{array}$$

It is easy to see that the SER for the LU for the proposed DM schemes is primarily affected by the information power splitting factor $\beta_{1}$, while the SER for the Eve is dependent on the information power splitting factor $\beta_{1}$ and the eavesdropper steering vector both.

### 5.2. Secrecy Rate

#### 5.2.1. Single-Carrier DM Scheme

Using Equation (17), the signal-to-interference-plus-noise ratio (SINR) of the LU for the *l*th transmit antenna array can be obtained by

(37) $$\begin{array}{c} \gamma_{l}^{sc,LU}=\frac{P_{s}\beta_{1}^{2}{\left(1-\xi \right)}^{2}}{\sigma^{2}}=r^{sc,LU}. \end{array}$$

Since the normalized narrow-band AWGN channel is assumed, by means of Equation (37), the achievable rate of the link from the *l*th transmit antenna array to the LU can be expressed as

(38) $$\begin{array}{c} R_{l}^{sc,LU}=\log_{2}\left(1+\gamma_{l}^{sc,LU}\right). \end{array}$$

In light of Equation (19), the SINR of the Eve for the *l*th transmit antenna array can be calculated by Equation (39).

(39) $$\begin{array}{c} \gamma_{l}^{sc,E}=\frac{P_{s}\beta_{1}^{2}|h^{H}\left(\theta_{el}\right)h\left(\theta_{dl}\right){|}^{2}}{P_{s}\beta_{1}^{2}\sum_{k=1,k\neq l}^{K}|h^{H}\left(\theta_{el}\right)h\left(\theta_{dk}\right){|}^{2}+P_{s}\beta_{2}^{2}\sum_{k=1}^{K}|h^{H}\left(\theta_{el}\right)T_{k}z_{k}{|}^{2}+\sigma^{2}}. \end{array}$$

According to Equation (39), the achievable rate of the link between the *l*th transmit antenna array and the Eve can be given by

(40) $$\begin{array}{c} R_{l}^{sc,E}=\log_{2}\left(1+\gamma_{l}^{sc,E}\right). \end{array}$$

Therefore, the secrecy rate of the proposed single-carrier DM scheme can be defined as [24]

(41) $$\begin{array}{c} R^{sc}=\sum_{l=1}^{K}{\left[R_{l}^{sc,LU}-R_{l}^{sc,E}\right]}^{†}. \end{array}$$

#### 5.2.2. Multi-Carrier DM Scheme

Likewise, according to Equation (26), the SINR of the LU for the *l*th transmit antenna array in the multi-carrier DM scheme can be calculated by

(42) $$\begin{array}{c} \gamma_{l}^{mc,LU}=\frac{P_{s}\beta_{1}^{2}}{\sigma^{2}}=r^{mc,LU}. \end{array}$$

By means of Equation (42), the achievable rate of the link from the *l*th transmit antenna array to the LU in the multi-carrier DM scheme can be written as

(43) $$\begin{array}{c} R_{l}^{mc,LU}=\log_{2}\left(1+\gamma_{l}^{mc,LU}\right). \end{array}$$

Using Equation (28), the SINR of the Eve for the *l*th transmit antenna array in the multi-carrier DM scheme can be derived as

(44) $$\begin{array}{c} \gamma_{l}^{mc,E}=\frac{P_{s}\beta_{1}^{2}|h^{H}\left(\theta_{e}\right)h\left(\theta_{dl}\right){|}^{2}}{P_{s}\beta_{2}^{2}|h^{H}\left(\theta_{e}\right)T_{l}z_{l}{|}^{2}+\sum_{k=1,k\neq l}^{K}|h^{H}\left(\theta_{e}\right)u_{k}{|}^{2}+\sigma^{2}}. \end{array}$$

Furthermore, from Equation (44), the achievable rate of the link between the *l*th transmit antenna array and the Eve in the multi-carrier DM scheme can be expressed as

(45) $$\begin{array}{c} R_{l}^{mc,E}=\log_{2}\left(1+\gamma_{l}^{mc,E}\right). \end{array}$$

Therefore, the secrecy rate of the proposed multi-carrier DM Scheme can be defined as [24]

(46) $$\begin{array}{c} R^{mc}=\sum_{l=1}^{K}{\left[R_{l}^{mc,LU}-R_{l}^{mc,E}\right]}^{†}. \end{array}$$

### 5.3. Robustness

In this section, the robustness of the proposed DM schemes about the impacts of imperfect estimation of the directions from all transmit antenna arrays to the LU is investigated.

In a general way, the implementation of DM is based on the assumption that the a priori directional information is known for the BS. However, in a practical DM system, there always exists an estimated error in the directional information even by adopting accurate direction of arrival (DOA) estimation methods (see, e.g., [46,47,48,49]) and in the range information even by using high-resolution range estimation algorithm (see, e.g., [50]). Therefore, the estimated angle error would result in angle mismatch including the transmit pre-coding vector, i.e., the excitation signal vector $v_{k}$ and the AN projection matrix $T_{k}$ for the *k*th transmit antenna array, which severely degrades the performance of the proposed DM schemes.

Here, the estimated directional angle of the *k*th transmit array can be expressed as

(47) $$\begin{array}{c} {\hat{\theta }}_{dk}=\theta_{dk}+\Delta \theta_{dk}, \end{array}$$

where $\Delta \theta_{dk}$ is the estimated directional angle error.

It is noted that the angle estimation errors impose negative impact not only on the precoding vector $v_{k}$, but also on the AN projection matrix $T_{k}$, while the architectures of the BS and the LU remain unchanged. For both proposed DM schemes, the precoding vector vector $v_{k}$ and the AN projection matrix $T_{k}$ with estimation errors should be rewritten as

(48) $$\begin{array}{c} {\hat{v}}_{k}=h\left({\hat{\theta }}_{dk}\right), \end{array}$$

and

(49) $$\begin{array}{c} {\hat{T}}_{k}=I_{N}-h\left({\hat{\theta }}_{dk}\right)h^{H}\left({\hat{\theta }}_{dk}\right), \end{array}$$

respectively.

According to Equation (48), it is easy to obtain that the normalization characteristic in Equation (5) will be affected by the estimation error. Likewise, from Equation (49), it is effortless to derive that the orthogonal property in Equation (7) will also be influenced when an estimation error exists, as analyzed in [51]. Simulations are provided in next Section to illustrate that the security can also be ensured on condition that the estimation errors of the desired directions are within an acceptable limit for the proposed DM schemes.

### 5.4. Comparisons for Multiple Antenna Arrays DM and Single Antenna Array DM Schemes

The comparisons for multiple antenna arrays DM and single antenna array DM schemes [7,8,9,10,11,12,13,14,15,16,17,18,19,20,21,22,23,24,25,26,27] are given in Table 1, from which the main features of the proposed DM schemes can be summarized as follows:

(1) Because the AN is inserted into the transmitting signals, both the pre-coding vector for the confidential information and the orthogonal projection matrix for the AN are required for all AN-aided DM schemes.

(2) Although multiple transmit antenna arrays are utilized in the proposed DM schemes, the computation complexity at the transmitter side doesn’t increase when transmitting *K* symbols each time. Obviously, the cost of the BS for the proposed schemes is more than that of the traditional single antenna array DM schemes.

(3) The proposed DM schemes can achieve precise transmission, i.e., projecting the confidential information to the LU located at the designated location. More importantly, transmission security of the proposed schemes can also be guaranteed even if the Eve is located close to the LU or the same direction as the LU.

As for the differences between the two proposed DM schemes, on the one hand, at the receiver side, the computation complexity of the single-carrier DM scheme is much higher than that of the multi-carrier DM scheme. On the other hand, the cost of the receiver for the proposed single-carrier DM scheme is much lower. Because the receive multi-beamforming method is adopted to recover the useful signal in the single-carrier DM scheme. In the multi-carrier DM scheme, the useful signals are obtained by using different band-pass filters. Therefore, the compromise of the computation complexity and the cost should be considered when choosing between these two different schemes to perform secure and precise wireless transmission.

## 6. Simulation Results and Discussions

**Figure 7:**
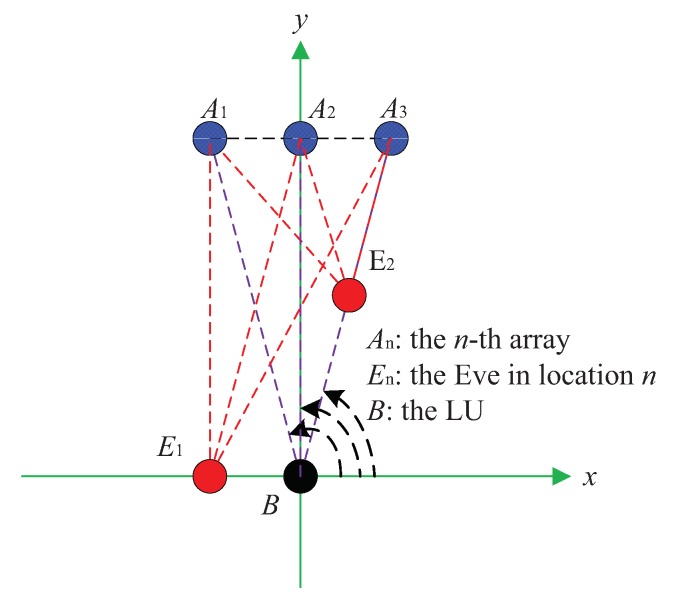
The locations of the LU, the potential Eve, and three transmit antenna arrays.

Three important metrics, namely the SER, secrecy rate, and the robustness of the proposed DM systems, were simulated. The detailed simulation parameters are provided in Table 2. Meanwhile, the locations of the LU, the potential Eve, and multiple transmit antenna arrays in the Cartesian coordinate system are shown in Figure 7.

To simplify the model and analysis, assuming that there exist K=3 transmit antenna arrays with N=21 antennas for each array. As mentioned in Table 1, cost, receiver complexity and transmitter complexity of the proposed DM schemes all depend on the number *K* of the ULA. Therefore, the choosing of the number *K* of the ULA is a compromise between the security performance and the cost in practical applications. To avoid grating lobe effect and mutual coupling between the array elements, the inter-element spacing is set one-half of a wavelength for each ULA [52,53,54,55,56,57,58,59,60,61]. For mm-wave system, applications for B5G and future wireless networks, the carrier frequencies can be set as 30 GHz and more. To distinguish different direction information for different antenna arrays, the antenna arrays should be away from each other, thus we set the inter-array spacing as far as 100λ. In the setup, one LU and one Eve in two potential locations are considered. In detail, for the first scenario, we assume for three transmit antenna arrays, the desired location *B* and eavesdropper $E_{1}$ are all located in different directions, where $\theta_{BA_{1}}=95.7392^{\circ }$, $\theta_{BA_{2}}=90^{\circ }$, $\theta_{BA_{3}}=84.2608^{\circ }$, $\theta_{E_{1}A_{1}}=90^{\circ }$, $\theta_{E_{1}A_{2}}=84.2608^{\circ }$, $\theta_{E_{1}A_{3}}=78.6346^{\circ }$, with the distance $d_{BA_{1}}=1000\lambda$, $d_{BA_{2}}=994.9874\lambda$, $d_{BA_{3}}=1000\lambda$, $d_{E_{1}A_{1}}=994.9874\lambda$, $d_{E_{1}A_{2}}=1000\lambda$, $d_{E_{1}A_{3}}=1014.9\lambda$. Likewise, for the second scenario, we assume for the third transmit antenna array, the desired location *B* and eavesdropper $E_{2}$ are located in the same direction, where $\theta_{BA_{1}}=95.7392^{\circ }$, $\theta_{BA_{2}}=90^{\circ }$, $\theta_{BA_{3}}=84.2608^{\circ }$, $\theta_{E_{2}A_{1}}=106.7787^{\circ }$, $\theta_{E_{2}A_{2}}=95.7932^{\circ }$, $\theta_{E_{2}A_{3}}=84.2608^{\circ }$, with the distance $d_{BA_{1}}=1000\lambda$, $d_{BA_{2}}=994.9874\lambda$, $d_{BA_{3}}=1000\lambda$, $d_{E_{2}A_{1}}=519.6152\lambda$, $d_{E_{2}A_{2}}=500\lambda$, $d_{E_{2}A_{3}}=500\lambda$. It is noted that all transmit antenna arrays in Figure 7 can also be distributed to other appropriate locations. Here, we just provide a setup case to evaluate the performance of the proposed DM schemes.

Since the maximal channel delay for different transmit antenna arrays is far less than symbol duration, all transmitted symbols can be thought of as being simultaneously received by the LU or the Eve.

### 6.1. SER

**Figure 8:**
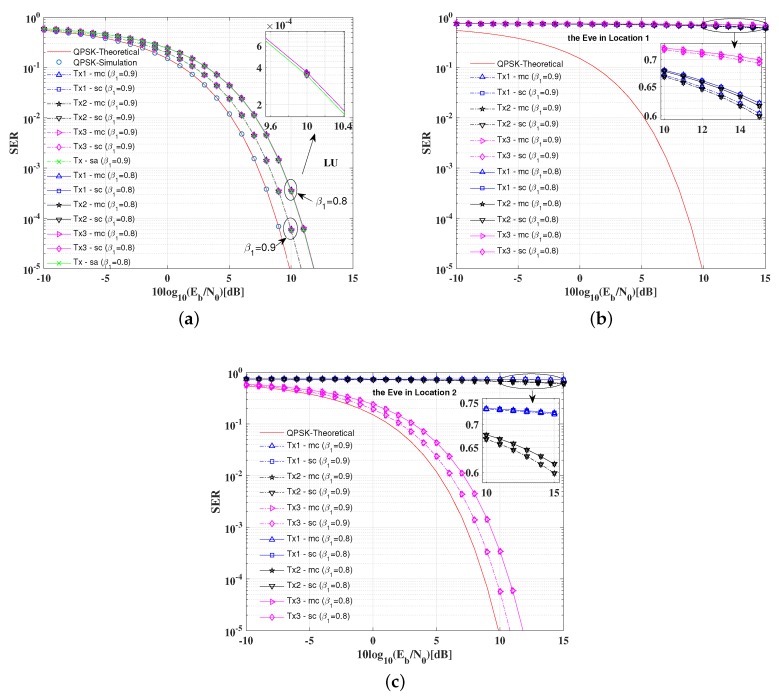
SER performance versus SNR per bit (dB) with various power allocation factors for the proposed DM schemes and the conventional DM scheme: (**a**) the LU; (**b**) the Eve in Location 1; and (**c**) the Eve in Location 2.

Figure 8 illustrates the SER performance of the LU and the Eve in two positions with various power allocation factors for the proposed multiple antenna arrays DM schemes and the conventional single antenna array DM schemes, where the baseband modulation modes are all set as QPSK, respectively.

Several conclusions can be summarized from Figure the results in Figure 8a: (1) For the LU, when the signal power allocation factor is set as $\beta_{1}=0.9$, to achieve the same SER level (e.g., $SER=10^{-4}$), the SNR per bit needed is approximately 1 dB less than that of the standard QSPK modulation, which indicates that the inserted AN will cause some degree of SNR loss. (2) Given a fixed SNR per bit (e.g., $10\log_{10}\left(E_{b/}N_{0}\right)=10\;\mathrm{dB}$), the SERs for the LU increase as the signal power allocation factors decrease. (3) The SER performance of the single antenna array DM schemes is the same as the multi-carrier multiple antenna arrays DM scheme, while the SER performance for the single-carrier multiple antenna arrays scheme is slightly worse than other two DM schemes.

From the results in Figure 8b, the SERs of three symbol streams for the Eve in Location 1 are approximate to the worst theoretical value, which indicates that the transmission security can be guaranteed when the Eve is not located in any desired directions.

From Figure the results in Figure 8c, the SERs of the symbol stream 1 and 2 for the Eve are as worse as the idea value, while SERs of the symbol stream 3 are as good as that of the LU, because the Eve is in the desired direction of the third transmit array. However, the transmission security can also be guaranteed because the confidential information received by the Eve is not intact.

**Figure 9:**
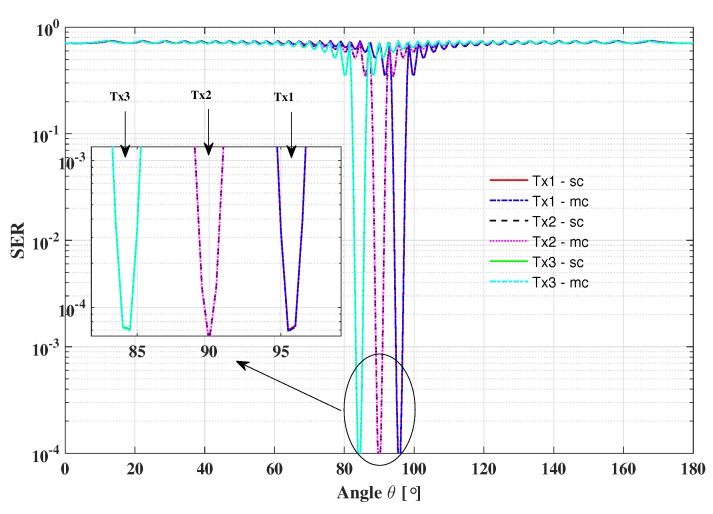
SER performance versus azimuth angle for the proposed DM schemes, where $\beta_{1}=0.9$ and $10\log_{10}\left(E_{b/}N_{0}\right)=10\;\mathrm{dB}$.

The SER performance versus azimuth angle for the two proposed DM schemes is shown in Figure 9. In this scenario, the baseband modulation is also set as QPSK, the SNR per bit is 10 dB, and the signal power splitting factor is set as $\beta_{1}=0.9$.

As we can see, the information beam-width ($SER=10^{-4}$) of the two proposed schemes for each symbol stream is almost the same. Meanwhile, the information beam-width ($SER=10^{-4}$) for different symbol streams is also the same because each transmitter can be regarded as a single antenna array DM transmitter. Obviously, only the receiver located at the intersection of three desired directions can recover the full confidential information. By contrast, the receiver located in other positions will be interfered by AN and the mixed signals. Therefore, the security of the proposed DM schemes can be hold even if part of the useful information is intercepted.

### 6.2. Secrecy Rate

**Figure 10:**
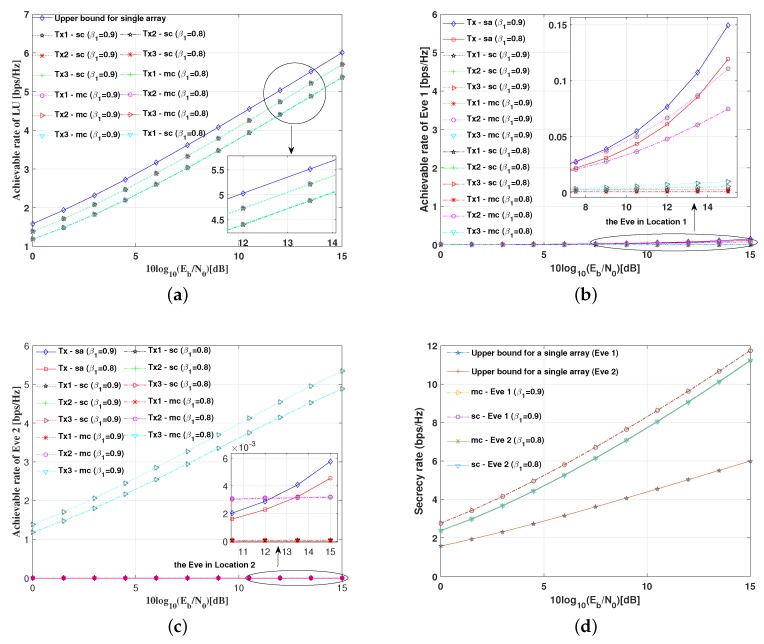
Achievable rate of the proposed DM schemes: (**a**) achievable rate of the LU versus SNR; (**b**) achievable rate of the Eve in Location 1 versus SNR; (**c**) achievable rate of the Eve in Location 2 versus SNR; and (**d**) secrecy rate performance of the proposed DM schemes.

Figure 10a demonstrates the achievable rate of the LU versus SNR per bit for the proposed DM schemes. As might have been expected, the achievable rate of the LU for each antenna array is almost at the same level. Owing to the introduction of AN, the achievable rates are lower than the upper bound of the achievable rate using a single antenna without AN. Especially, the achievable rates of the LU decrease as the information power splitting factors decrease.

Figure 10b,c shows the achievable rate of the Eve at two different potential positions. As expected, the achievable rates are approximate to 0 bps/Hz when the Eve is not in any desired directions. Once the Eve is in one of the desired directions, the achievable rates for the Eve are just a little lower than that of the LU.

Furthermore, Figure 10d illustrates the secrecy rate versus SNR per bit for the proposed DM schemes and the traditional single antenna array DM schemes. Whatever value the SNR per bit and the power allocation factor $\beta_{1}$ take, the secrecy rates of the proposed DM schemes are much higher than those of traditional single antenna array transmission schemes with the same fixed total transmitting power. As expected, the secrecy rates of the proposed DM schemes are almost twice those of the traditional single antenna array schemes due to the use of multiple antenna array simultaneously. By contrast, the secrecy rates are always positive wherever the Eve is located. Therefore, the proposed DM schemes are very secure and reliable.

### 6.3. Robustness

**Figure 11:**
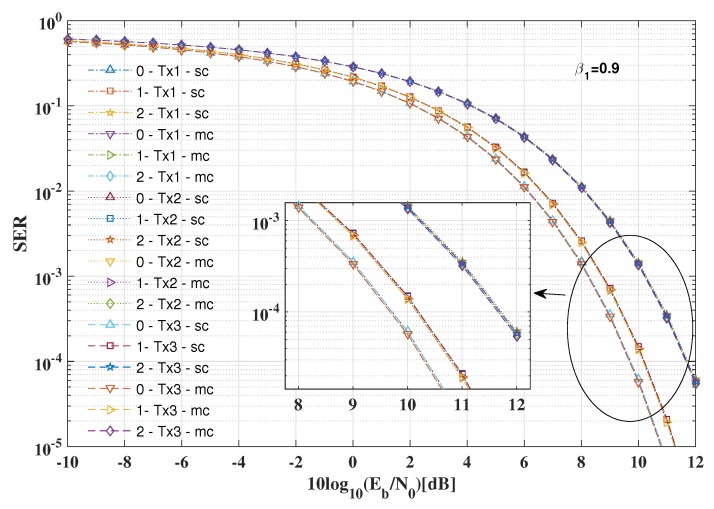
Robustness of the proposed DM schemes with various estimation errors of the desired directions.

The SER performance versus SNR per bit with different imperfect estimation errors of the desired directions for three transmit antenna arrays is displayed in Figure 11. For clarity, the signal power allocation factor is set as $\beta_{1}=0.9$, and only the curves for the first antenna array are analyzed, from which the same conclusions can also hold for other two antenna arrays. It is easy to observe that there is only approximately 0.5 dB loss of SNR per bit when the estimated direction error is $\Delta \theta =1^{\circ }$ for a fixed SER performance (e.g., $SER=10^{-4}$). As long as the estimated direction errors for one desired direction are within $\Delta \theta =1^{\circ }$, at most 0.5 dB extra SNR per bit is needed to realize the same SER as the ideal case. Therefore, to achieve secure and precise transmission for the LU, the estimated direction errors for all antenna arrays should be less than $\Delta \theta =1^{\circ }$.

## 7. Conclusions

Based on a multiple antenna arrays transmission model at the transmitter side for DM, where all signals from multiple antenna arrays are mixed at the receiver side, two secure and precise AN-aided DM schemes with single carrier and multiple carriers were proposed, respectively. Then, the SER, secrecy rate, and the robustness performance of the proposed schemes were analyzed and simulated. Simulation results demonstrate the advantages of precise transmission and neighbor security for the proposed schemes over the traditional AN-aided DM scheme using a single antenna array. Meanwhile, it is of considerable interest to extend to a multi-user and multi-eavesdropper DM system over multi-path fading channels. Moreover, the robust DM designs for the proposed schemes are also well worth considering. One future work is to investigate and validate the security performance of the proposed DM schemes in practical LOS communication scenarios.

## Figures and Tables

**Table 1:** Comparisons for multiple antenna arrays DM and single antenna array DM schemes.

Items	Single-Carrier DM	Multi-Carrier DM	Single Array DM [7,8,9,10,11,12,13,14,15,16,17,18,19,20,21,22,23,24,25,26,27]
Multiple antenna arrays	Yes	Yes	No
Multiple carriers	No	Yes	No
Artificial noise	Yes	Yes	Yes
Precoding vector	Yes	Yes	Yes
Orthogonal matrix	Yes	Yes	Yes
Receiver complexity ${}^{1}$	$O(\varsigma_{1})$	$O(2\varsigma_{2})$	$O(2\varsigma_{2})$
Transmitter complexity	$O(\varsigma_{2})$	$O(\varsigma_{2})$	$O(\varsigma_{2})$
Cost	High	Higher	Low
Precise transmission ${}^{2}$	Yes	Yes	No
Neighbor security ${}^{3}$	Yes	Yes	No

${}^{1}$$\varsigma_{1}=\left(2NK+K^{2}\right)\left(K-1\right)+NK{\left(K-1\right)}^{2}+K{\left(K-1\right)}^{2}$ and $\varsigma_{2}=KN^{2}+NK$. ${}^{2}$ The case where the confidential information is transmitted to a preassigned position. ${}^{3}$ The scenario where the Eve is close to the information beam-width region [7].

**Table 2:** Simulation parameters.

Parameters	Value
Number of ULA, *K*	3
Number of ULA elements, *N*	21
Single carrier frequency, $f_{c}$	30 GHz
Inter-array spacing, *D*	100λ
Inter-element spacing, *d*	0.5λ
Multiple carrier frequencies, $f_{c1}$, $f_{c2}$, $f_{c3}$	30 GHz, 30.1 GHz, 30.2 GHz
Total transmit power per symbol, $P_{s}$	1
Number of LU	1
Number of Eve	1
Location of the 1st array ${}^{*}$, $\left(r_{1},\theta_{1}\right)$	$\left(1000\lambda ,95.7392^{\circ }\right)$
Location of the 2nd array ${}^{*}$, $\left(r_{2},\theta_{2}\right)$	$\left(994.9874\lambda ,90^{\circ }\right)$
Location of the 3rd array ${}^{*}$, $\left(r_{3},\theta_{3}\right)$	$\left(1000\lambda ,84.2608^{\circ }\right)$
Location 1 for the Eve ${}^{*1}$, $\left(r_{1}^{E},\theta_{1}^{E}\right)$	$\left(100\lambda ,180^{\circ }\right)$
Location 2 for the Eve ${}^{*2}$, $\left(r_{1}^{E},\theta_{1}^{E}\right)$	$\left(500\lambda ,84.2608^{\circ }\right)$
Modulation mode	QPSK
Information rate	20 Mbps

${}^{*}$ The location of the LU is regarded as the reference point. ${}^{1}$ The case where the Eve is not in the same direction as the LU for any arrays. ${}^{2}$ The case where the Eve is located in the same direction as the LU for the 3rd array.

## Appendix A

**Proof** **of Equation (15).** From Equation (11), we can rewrite Equation (15) as

(A1) $$\begin{array}{cc} y_{l} & =w_{l}^{H}\left[\sum_{k=1}^{K}\left(\beta_{1}\sqrt{P_{s}}h\left(\theta_{dk}\right)s_{k}+\beta_{2}\sqrt{P_{s}}T_{k}z_{k}\right)+n^{{}^{\prime }}\right]\\ \; & =\underset{p1}{\underset{︸}{w_{l}^{H}\beta_{1}\sqrt{P_{s}}h\left(\theta_{dl}\right)s_{l}}}+\underset{p2}{\underset{︸}{w_{l}^{H}\sum_{k=1,k\neq l}^{K}\beta_{1}\sqrt{P_{s}}h\left(\theta_{dk}\right)s_{k}}}+\underset{p3}{\underset{︸}{w_{l}^{H}\sum_{k=1}^{K}\beta_{2}\sqrt{P_{s}}T_{k}z_{k}}}+\underset{p4}{\underset{︸}{w_{l}^{H}n^{\prime }}}. \end{array}$$

Here, for p1, using Equations (13) and (15), we have

(A2) $$\begin{array}{cc} p1 & ={\left(P_{A}^{⊥}h\left(\theta_{dl}\right)\right)}^{H}\beta_{1}\sqrt{P_{s}}h\left(\theta_{dl}\right)s_{l}\\ \; & =h^{H}\left(\theta_{dl}\right){\left(P_{A}^{⊥}\right)}^{H}\beta_{1}\sqrt{P_{s}}h\left(\theta_{dl}\right)s_{l}\\ \; & =\beta_{1}\sqrt{P_{s}}s_{l}\left[h^{H}\left(\theta_{dl}\right)h\left(\theta_{dl}\right)-h^{H}\left(\theta_{dl}\right)A{\left(A^{H}A\right)}^{-1}A^{H}h\left(\theta_{dl}\right)\right]\\ \; & =\beta_{1}\sqrt{P_{s}}s_{l}\left(1-\xi \right). \end{array}$$

For p2, using Equation (16), we have

(A3) $$\begin{array}{c} p2=\beta_{1}\sqrt{P_{s}}s_{k}\sum_{k=1,k\neq l}^{K}w_{l}^{H}h\left(\theta_{dk}\right)=0. \end{array}$$

Substituting Equations (14) and (15) into p3 in Equation (A1) and using Equation (7), we have

(A4) $$\begin{array}{cc} p3 & =\beta_{2}\sqrt{P_{s}}\sum_{k=1}^{K}w_{l}^{H}T_{k}z_{k}\\ \; & =\beta_{2}\sqrt{P_{s}}\sum_{k=1}^{K}\left[h^{H}\left(\theta_{dl}\right)T_{k}z_{k}-h^{H}\left(\theta_{dl}\right)A{\left(A^{H}A\right)}^{-1}A^{H}\right)T_{k}z_{k}]\\ \; & =\beta_{2}\sqrt{P_{s}}\sum_{k=1}^{K}[0z_{k}-h^{H}\left(\theta_{dl}\right)A{\left(A^{H}A\right)}^{-1}0z_{k}]\\ \; & =0. \end{array}$$

Therefore, by Equations (A2)–(A4), Equation (A1) can be transformed into

(A5) $$\begin{array}{c} y_{l}=\beta_{1}\sqrt{P_{s}}s_{l}\left(1-\xi \right)+n_{l}. \end{array}$$

The proof is completed. □
